# Life history constraints explain negative relationship between fish productivity and dissolved organic carbon in lakes

**DOI:** 10.1002/ece3.3108

**Published:** 2017-07-03

**Authors:** Nicola Craig, Stuart E. Jones, Brian C. Weidel, Christopher T. Solomon

**Affiliations:** ^1^ Dept. of Natural Resource Sciences McGill University Ste. Anne de Bellevue QC Canada; ^2^ Dept. of Biological Sciences University of Notre Dame Notre Dame IN USA; ^3^ U.S. Geological Survey Great Lakes Science Center Lake Ontario Biological Station Oswego NY USA; ^4^ Cary Institute of Ecosystem Studies Millbrook NY USA

**Keywords:** Centrarchidae, humic waters, lake browning, life history trade‐offs, reproductive output, terrestrial subsidies, water color

## Abstract

Resource availability constrains the life history strategies available to organisms and may thereby limit population growth rates and productivity. We used this conceptual framework to explore the mechanisms driving recently reported negative relationships between fish productivity and dissolved organic carbon (DOC) concentrations in lakes. We studied populations of bluegill (*Lepomis macrochirus*) in a set of lakes with DOC concentrations ranging from 3 to 24 mg/L; previous work has demonstrated that primary and secondary productivity of food webs is negatively related to DOC concentration across this gradient. For each population, we quantified individual growth rate, age at maturity, age‐specific fecundity, maximum age, length‐weight and length‐egg size relationships, and other life history characteristics. We observed a strong negative relationship between maximum size and DOC concentration; for instance, fish reached masses of 150 to 260 g in low‐DOC lakes but <120 g in high‐DOC lakes. Relationships between fecundity and length, and between egg size and length, were constant across the DOC gradient. Because fish in high‐DOC lakes reached smaller sizes but had similar fecundity and egg size at a given size, their total lifetime fecundity was as much as two orders of magnitude lower than fish in low‐DOC lakes. High DOC concentrations appeared to constrain the range of bluegill life history strategies available; populations in high‐DOC lakes always had low initial growth rates and high ages at maturity, whereas populations in low‐DOC showed higher variability in these traits. This was also the case for the intrinsic rates of natural increase of these populations, which were always low at the high end of the DOC gradient. The potentially lower capacity for fish populations in high‐DOC lakes to recover from exploitation has clear implications for the sustainable management of recreational fisheries in the face of considerable spatial heterogeneity and ongoing temporal change in lake DOC concentrations.

## 1 INTRODUCTION

Resource availability is a major driver for selection of various life history strategies in organisms and can ultimately affect population growth and productivity (Begon, Townsend, & Harper, 2006; MacArthur & Wilson, 1967; Wilbur, Tinkle, & Collins, 1974). When resources are limited, less energy is available for growth and reproduction, necessitating reduced allocation to one or the other, with implications for lifetime reproductive output and fitness (Fisher, 1930; Stearns & Koella, 1986). Relative to more productive environments, organisms in unproductive environments may grow more slowly and allocate their limited energy to growth for a longer time until they become large enough to produce sufficient offspring (MacArthur & Wilson, 1967; Pianka, 1970). In such cases, organisms are likely to mature later than those in more productive environments, which can grow faster and get larger quicker. Alternatively, organisms in such environments may mature at a similar age but smaller size, again reducing the capacity for reproductive output (Stearns & Koella, 1986). Understanding how life history traits are affected by resource limitation is important in predicting how populations may react to shifts in resource availability.

In lake ecosystems, high levels of dissolved organic carbon (DOC) reduce ecosystem productivity and resource availability through the limitation of light and habitat availability (Craig, Jones, Weidel, & Solomon, 2015; Karlsson et al., 2009; Kelly, Solomon, Weidel, & Jones, 2014). Terrestrially derived DOC, which makes up the majority of the DOC pool in many lakes, is flushed in from the surrounding landscape and stains the water, such that high‐DOC lakes have a dark brown color (Jones, 1992; Wilkinson, Pace, & Cole, 2013). This darkening of the water has physical and biological effects on lakes by reducing light and heat penetration and thus reducing thermocline depths, restricting the area of well‐oxygenated epilimnion (Read & Rose, 2013; Wetzel, 2001). As a result of these effects, primary production is limited due to the diminished light climate (Ask et al., 2009; Godwin, Jones, Weidel, & Solomon, 2014) and secondary production is limited due to both reduced primary production and area of suitable habitat (Craig et al., 2015; Karlsson et al., 2009; Kelly et al., 2014). DOC‐mediated reductions in resource and habitat availability will likely affect fish life history strategies in ways that are currently poorly understood (Solomon et al., 2015; Stasko, Gunn, & Johnston, 2012). This is of particular concern because DOC concentrations have been rising in northern hemisphere freshwaters over the past two decades (Evans, Monteith, & Cooper, 2005; Monteith et al., 2007; Solomon et al., 2015) and are also spatially heterogeneous, spanning wide gradients across the landscape (Hanson, Carpenter, Cardille, Coe, & Winslow, 2007).

Recent studies have shown that high levels of DOC can have a negative effect on fish production and abundance (Finstad, Helland, Ugedal, Hesthagen, & Hessen, 2014; Karlsson et al., 2009). Both these studies were based on lake surveys over a DOC gradient in Scandinavia and estimated fish production based on gillnet catch‐per‐unit‐effort. Karlsson et al. (2009) found a negative relationship between production and DOC, and Finstad et al. (2014) found a unimodal relationship with low fish productivity at very low, and high DOC concentrations. In both cases, the major mechanism proposed for a reduction in fish productivity at high DOC concentrations was reduced basal resource availability due to lower light climates in darker lakes. However, neither study considered how this resource limitation may manifest in fish life history traits, leading to lower productivity in terms of growth or reproduction.

In this study, we quantified how fish life histories varied across eleven lakes that ranged widely in DOC concentration, by estimating initial growth, reproductive output, and age at maturity of female bluegill (*Lepomis macrochirus*; Centrarchidae). In high‐DOC (dark) environments, fish populations are likely to be resource‐limited and have less surplus energy available to allocate to growth and reproduction; we predicted that this may lead to slower somatic growth, smaller maximum size, later age at maturity, and thus a lower overall fecundity relative to populations in low‐DOC (clear) lakes (Charnov, 1993; Roff, 1983; Stearns & Koella, 1986).

## 2 METHODS

### 2.1 Study system and data collection

Our study focused on bluegill as they are widely distributed over gradients of DOC in North America, have variable life history strategies, and are a popular sport fish of economic value (Drake, Claussen, Philipp, & Pereira, 1997). Fish were collected from eleven lakes near the Wisconsin–Michigan border, including the University of Notre Dame Environmental Research Centre and the surrounding area of Vilas County, Wisconsin. The lakes spanned broad gradients of size, nutrients and DOC (Table 1), and had similar fish assemblages with top predators including largemouth bass (*Micropterus salmoides*), northern pike (*Esox lucius*), and walleye (*Sander vitreus*).

**Table 1:** Summary of lake parameters for the eleven lake survey during the study period. DOC is dissolved organic carbon. Standard deviation is in parentheses

Lake	Area (ha)	Max depth (m)	DOC (mg/L)	Total phosphorus (μg/L)
Big Arbor Vitae	433	12.5	3.1 (0.6)	29.1 (22.4)
Crampton	25.9	18.5	5.0 (0.9)	9.4 (2.7)
Allequash	168.4	8	5.7 (1.3)	17.7 (4.6)
Erickson	44.5	5.5	6.2 (0.7)	19.6 (16.7)
Bay	67.3	12.2	7.4 (1.7)	12.4 (5.7)
Deadwood	9.7	8.8	9.7 (2.8)	12.4 (3.6)
Tenderfoot	194.2	9.14	12.0 (2.5)	15.5 (3.1)
Birch	204.7	13.7	12.5 (1.1)	12.1 (5.5)
McCullough	89.4	8.2	14.3 (1.7)	14.0 (7.2)
Red Bass	10.9	6.7	18.9 (2.8)	45.0 (40.8)
Hummingbird	0.8	7.6	24.5 (5.2)	20.0 (7.4)

Bluegill were collected just before spawning (May–June) in 2013 and 2014, with the exception of Erickson Lake and Birch Lake which were only sampled in 2013. As bluegill are primarily a littoral species, particularly in spring and early summer (Becker, 1983), fish were collected using littoral fyke nets set overnight (2–10 net nights per lake per year), with the exception of McCullough where we were restricted to angling. Fyke nets have shown to be a reliable method to sample bluegill populations, yielding similar results to various other gears (Bethke & Staples, 2015; Fischer & Quist, 2014). For each sampling occasion, one 1‐m² square net (12‐m leader, no wings, 3‐m‐long car with 5 sections), and one 1‐m‐diameter round net (12‐m leader, two wings of 6 m, 2.5‐m‐long car with 4 sections) were set (both with a stretched mesh size of 1 inch, and 1‐m‐high leaders). Nets were set in each lake on subsequent nights until we had collected a sufficient number and size range of bluegill. We collected a wide range of sizes (from 75 mm to maximum size) in order to estimate size at maturity, maximum size, and the relationship between gonad mass and fish size. Catch‐per‐unit‐effort (CPUE) was estimated as the number of fish caught per hour of each net set (again with the exception of McCullough).

Fish were euthanized in a tricaine methane sulfonate (MS 222) solution and were frozen until they could be dissected. Once thawed, the fish were measured (mm) and weighed (g) and then the gonads were removed and weighed whole on a microbalance (g). Otoliths were also removed at this point. In 2014, we measured egg size and abundance for female fish (see Hunter, Lo, & Leong, 1985). We stored the gonads in formalin (1:10 formaldehyde dilution) until analysis. Each gonad was removed from the formalin, reweighed, and three replicate subsamples were taken and weighed. Each subsample was then placed on a microscope slide, and two drops of glycerol were applied to help separate the eggs. The number of mature eggs per subsample was counted under a stereo microscope, and then this was scaled up to estimate the number of mature eggs for the whole gonad. A photograph was also taken of each slide using a digital microscope camera, and egg widths were measured using ImageJ software (National Institutes of Health, USA).

The ages of a subset of fish were determined using otoliths in order to understand the age structure of the populations and to estimate age at maturity. Between 27 and 67 otoliths were analyzed per lake (mean = 44, see Appendix S1 for full details of sample sizes for various analyses). Sagittal otoliths were mounted in resin, and a transverse section (~300 μm thick) was cut through the otolith origin using a pair of diamond blades and a low‐speed IsoMet saw (Buehler, USA). Sections were polished with successively finer polishing pads and adhered to slides, and annuli were interpreted and counted under a compound microscope. Otoliths were interpreted by two individuals, and assigned ages were compared; if agreement on age could not be reached, the otolith was removed from analysis.

### 2.2 Environmental variables

Water chemistry samples and temperature profiles were collected twice during summer 2013 and three times during summer 2014. Water samples were taken using a Van Dorn bottle at three points in the epilimnion (bottom, middle, top) after which the pooled samples were filtered through 0.7‐μm GF/F filters, and DOC concentrations were measured using a Shimadzu TOC‐V total organic carbon analyzer (Shimadzu Scientific Instruments, Kyoto, Japan). Total phosphorous was measured using a colorimetric assay following a persulfate digestion (Menzel & Corwin, 1965). Chlorophyll *a* was measured through a fluorometric method after a methanol extraction (Welschmeyer, 1994). The temperature profiles were taken with a YSI Pro 20 polarigraphic sensor (Yellow Springs Instruments, U.S.A.), and thermocline depth was calculated as the point at which the change in temperature with depth was most rapid.

### 2.3 Growth, maximum age, and size

Initial growth rates (ω; mm/year) were estimated for each population by fitting the Gallucci and Quinn (1979) parameterization of the von Bertalanffy growth model to the length at age data for females in each lake, via maximum likelihood with a Gamma likelihood (Appendix S2, Fig. 2a). Confidence intervals for this parameter were estimated by quadratic approximation from the information matrix (Bolker, 2008).

Maximum length, mass, and age in each lake were estimated as the 95^th^ percentile of the size and age distributions, to avoid bias due to rare fish of extreme age or size. For maximum age, we first estimated the age of each captured fish from its size, using the lake‐specific age–mass relationship (Appendix S2, Fig. 2b). Maximum age was estimated in this way rather than from the subset of fish that were directly aged because that subset was a nonrandom sample intended to evenly span the entire size range in each lake. Both male and female fish were included in maximum size and age estimates because size at age was similar between the sexes and sex could not always be determined for bluegill released back into the field (Appendix S2, Fig.2a).

### 2.4 Size and age at maturity

Size at maturity was estimated using a broken‐stick regression of gonad mass on fish length (Appendix S2, Fig.2c, Bolker, 2008). The slope of gonad mass on length was set to zero for the first piece of the regression (immature fish), and the remaining parameters were estimated by maximum likelihood using a Gamma likelihood. The estimated size at maturity was converted to age at maturity using the lake‐specific age–length relationships. There are two lakes for which this model did not fit well. Tenderfoot Lake had few data points for immature fish and a shallow slope for mature fish which resulted in an estimated size at maturity that is probably too low. Also, no immature fish were collected in Deadwood Lake, and so the intercept of immature gonad mass was fixed as an average of the other lakes (0.43 g ± 0.07 standard error).

### 2.5 Fecundity and intrinsic rate of increase

Fecundity was estimated in two different manners: the potential lifetime fecundity of an individual if they reach maximum age, and the realized lifetime fecundity which incorporates the probability of mortality.

Potential lifetime fecundity for females in each lake was calculated based on lake‐specific relationships between age and body mass, body mass and gonad mass, and gonad mass and number of eggs, summed between the lake‐specific age at maturity and maximum age (Appendix S2, Figs 2b, 2d, and 2e). For Birch and Erickson lakes, we did not have data on the gonad mass to egg number relationship, so we used the across‐lake average in our calculations for these two lakes which did not vary over the DOC gradient (see 3).

Realized lifetime fecundity (R_0_), i.e., the mean number of eggs produced by an average individual accounting for mortality, was estimated using the formula:

$$R_{0}=\sum l_{x}m_{x}$$

where l_x_ is the proportion of the population surviving to age x, and m_x_ is the number of eggs produced at age x (Connell, 1970). Mortality was estimated from the fitted Von Bertalanffy growth function for each lake (Jensen, 1996). We also used the R_0_ estimates to calculate the intrinsic rate of natural increase, *r*, which is the change in population size per individual per unit time (*T*, in this case average generation time):

$$r=\text{ln}\;R_{0}/T$$

### 2.6 Testing effect of DOC on life history parameters

Linear regression models were used to test for effects of DOC concentration on life history parameters, and other potentially confounding lake characteristics. We included a random lake effect when the dependent variable was observed at the level of individual fish. These analyses were conducted in R using the lm function, and lme4 package (Bates, Maechler, Bolker, & Walker, 2015, R Core Team 2014).

## 3 RESULTS

**Figure 1:**
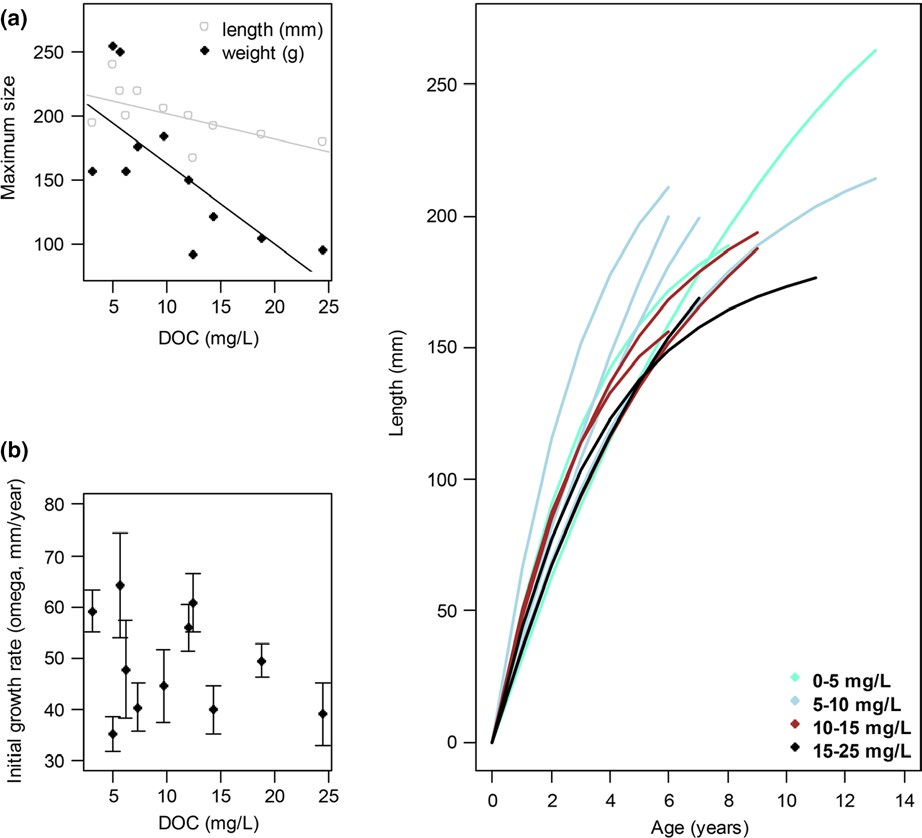
a) 95^th^ percentile of bluegill maximum length and mass regressed with DOC concentration (length: *F*_1,9_ = 5.73, *R* ^2^ = 0.39, *p* = .04; mass: *F*_1,9_ = 9.99, *R* ^2^ = 0.53, *p* = .01), b) Von Bertalanffy Gallucci–Quinn initial growth rate (ω, mm per year) regressed with DOC concentration (*F*_1,9_ = 0.76, *R* ^2^ = 0.08, *p* = .4), c) Von Bertalanffy length–age growth curves for bluegill for each lake; lines are color‐coded according to DOC concentration

**Figure 2:**
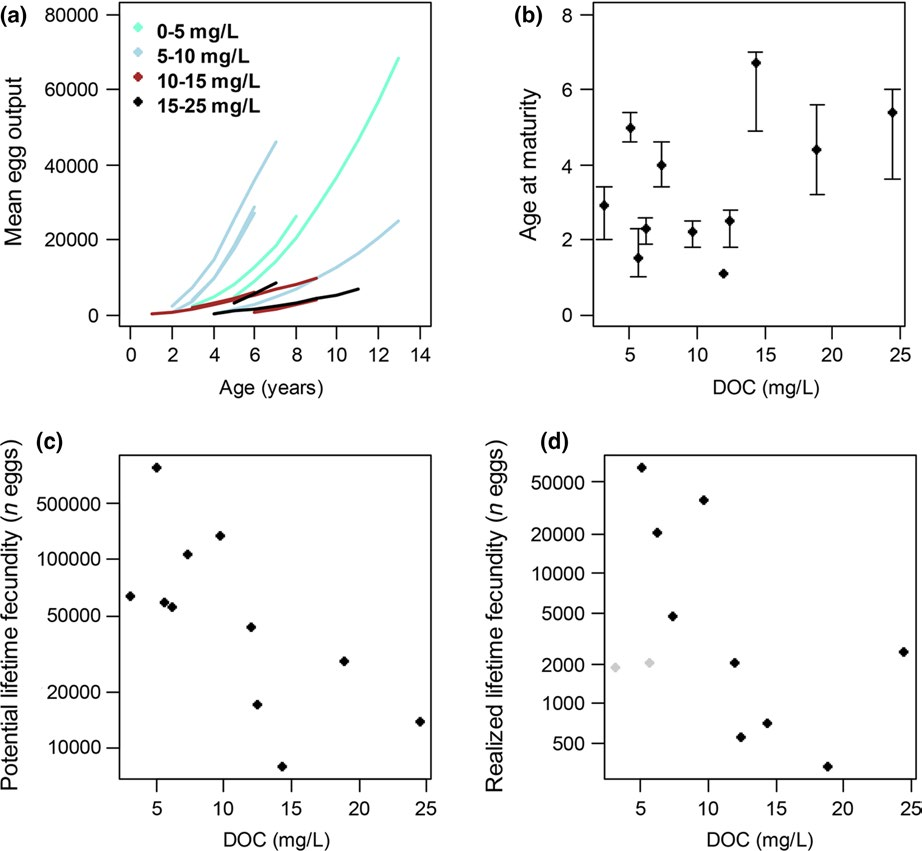
a) Mean age‐specific egg output of female bluegill. Each line indicates the relationship for one lake and extends from the age of maturity to maximum age. Lines are color‐coded according to the DOC concentration of the lake. b) Female bluegill age at maturity as a function of DOC (*F*_1,9_ = 2.26, *R* ^2^ = 0.2, *p* = .17). Error bars are 95% confidence intervals. c) Lifetime potential fecundity for female bluegills as a function of DOC (log(potential fecundity) vs DOC,* F*_1,9_ = 8.11, *R* ^2^ = 0.47, *p* = .02). d) Lifetime realized fecundity (i.e., average egg number per age accounting for mortality) of female bluegills as a function of DOC (log(realized fecundity) vs DOC,* F*_1,9_ = 2.75, *R* ^2^ = 0.2, *p* = .13). This relationship improved when the heavily fished lakes (Big Arbor Vitae and Allequash—gray points) were removed (*F*_1,7_ = 5.16, *R* ^2^ = 0.42, *p* = .06)

Bluegill in clear lakes attained larger sizes than those in darker lakes (Fig. 1). The 95^th^ percentiles of both length and mass were negatively related to DOC concentration (Fig. 1a, Appendix S2, Figs 2h, 2i; length: *F*_1,9_ = 5.73, *R* ^2^ = 0.39, *p* = 0.04; mass: *F*_1,9_ = 9.99, *R* ^2^ = 0.53, *p* = .01). The differences in maximum length and especially mass between clear and dark lakes were substantial; for instance, at the lower end of the DOC gradient, the 95th percentile of the largest fish sampled was generally between 150 and 260 g, whereas at the higher end the largest fish were <120 g. Initial growth rate was not significantly related to DOC concentration, although growth was slow in the darkest lakes and sometimes fast in clear lakes (Fig. 1b; *F*_1,9_ = 0.76, *R* ^2^ = 0.08, *p* = .4). Instead, fish in clear lakes apparently achieved large maximum size either by sustaining high growth rates for a shorter period of time, or by growing slowly for many years (Fig. 1c). Mass was strongly related to length (linear mixed effects (LME) slope = 0.99 ± 0.02), and this relationship was fairly constant across the DOC gradient (effect of DOC: LME slope = −0.04 ± 0.06).

**Figure 3:**
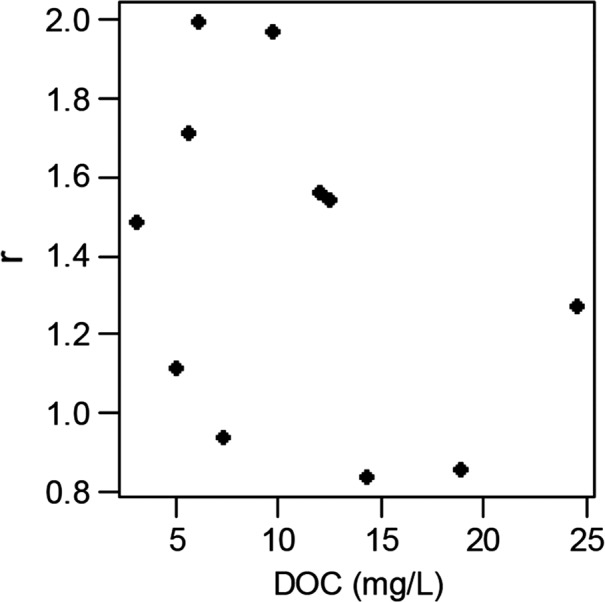
The intrinsic rate of natural population increase (r) plotted against DOC concentration (*F*_1,9_=1.45, *R* ^2^=0.14, *p* = .26)

Because fish in clearer lakes reached larger sizes, they also had much greater fecundity than those in dark lakes (Fig. 2). Age‐specific fecundity was positively related to fish size, and this relationship did not vary among lakes as a function of DOC concentration (LME slope of egg number on length: 0.73 ± 0.3, DOC slope: −0.01 ± 0.24, Appendix S2, Fig. 2f). Fecundity therefore increased much more quickly with age in clear lakes than in dark ones (LME DOC slope for log‐log regression: −1.1 ± 0.5; Fig. 2a). Egg size was not related to fish size except in Red Bass Lake, nor was it related to DOC concentration (LME length slope: 0.3 ± 0.3, DOC slope: −0.16 ± 0.24, Appendix S2, Fig. 2g). Populations with high initial growth rates matured at earlier ages (*F*_1,9_ = 8.5, *R* ^2^ = 0.48, *p* = .02). Given the relationship between initial growth and DOC (Fig. 1b), this meant that populations in darker lakes tended to mature later, and often at larger sizes, but that there was no significant linear relationship between DOC and age or size at maturity (age: *F*_1,9_ = 2.26, *R* ^2^ = 0.2, *p* = .17, Fig. 2b; size: *F*_1,9_ = 0.22, *R* ^2^ = 0.02, *p* = .6). Reproductive lifespan, which ranged from 3 to 10 years, was also not related to DOC concentration (*F*_1,9_ = 1.21, *R* ^2^ = 0.12, *p* = .3). Differences in age at maturity and especially age‐specific fecundity translated into large differences in lifetime potential fecundity, which was strongly negatively related to DOC concentration (Fig. 2c; *F*_1,9_ = 8.11, *R* ^2^ = 0.47, *p* = .02). Realized fecundity was also negatively related to DOC (Fig. 2d; *F*_1,8_=6.7, *p* = .03), but only after we accounted for the effects of fishing‐induced mortality by including an index of recreational fishing pressure in the analysis, which also was negatively related to lifetime fecundity (*F*_1,8_=13.8, *p* = .006). The intrinsic rate of natural increase was variable at low DOC concentrations and tended to be low at high DOC concentrations, although there was no significant linear relationship (Fig. 3; *F*_1,9_ = 1.45, *R* ^2^ = 0.14, *p* = .26).

**Figure 4:**
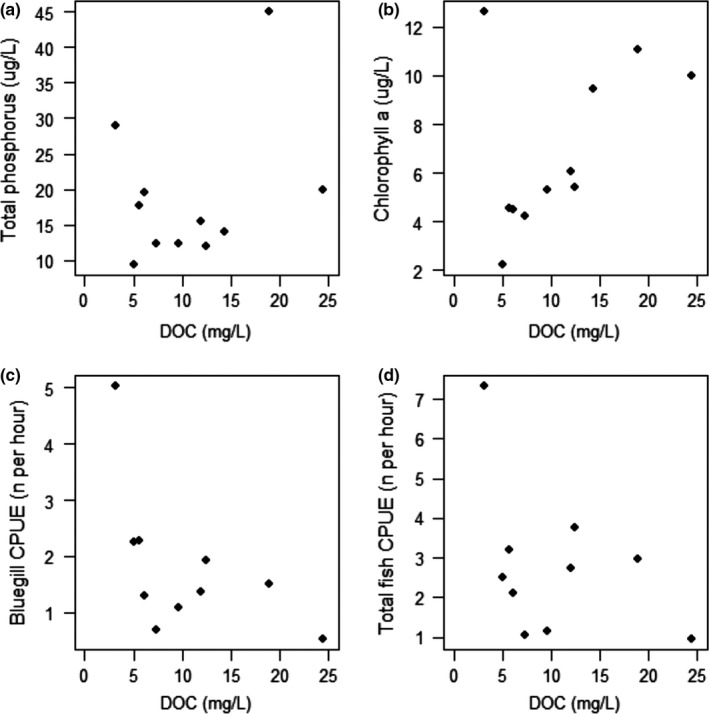
Relationships between DOC and a) total phosphorous, b) chlorophyll *a*, c) bluegill CPUE, and d) total fish CPUE

We also tested for the effects of potentially confounding variables on DOC which may affect fish life history strategies. There was no relationship between DOC and both bluegill, and total fish CPUE, a proxy for density, although CPUE was generally lower in the darker lakes (bluegill: *F*_1,8_ = 3.54, *R* ^2^ = 0.31, *p* = .1; all fish: *F*_1,8_ = 1.63, *R* ^2^ = 0.17, *p* = .24, Fig. 4c,d). We did, however, find a positive relationship between bluegill/total fish CPUE and thermocline depth, a proxy for habitat availability (bluegill: *F*_1,8_ = 14.58, *R* ^2^ = 0.65, *p* < .01; all fish: *F*_1,8_ = 15.38, *R* ^2^ = 0.66, *p* < .01). Thermocline depth was also significantly negatively related to DOC concentration (*F*_1,9_ = 11.52, *R* ^2^ = 0.56, *p* < .01). Variables representing basal nutrient/resource availability were not related to DOC (TP: *F*_1,9_ = 0.92, *R* ^2^ = 0.09, *p* = .36; Chl: *F*_1,9_ = 2.36, *R* ^2^ = 0.21, *p* = .16, Fig. 4a,b), although chlorophyll *a* content tended to increase along the gradient, and were significantly related when a major outlier was removed (Big Arbor Vitae, *F*_1,8_ = 34.6, *R* ^2^ = 0.81, *p *≤ .001).

## 4 DISCUSSION

### 4.1 The effects of DOC‐induced resource limitation on fish life histories

Our results demonstrate that life history mechanisms, such as reductions in size at age and maximum size leading to reduced reproductive output, could explain the negative effect of DOC on fish productivity. Fish in darker lakes are resource‐limited and thus appear to have less surplus energy to allocate to growth and reproduction (Craig et al., 2015; Diana, 1987; Karlsson et al., 2009; Lester, Shuter, & Abrams, 2004). Gonad mass at a given size does not change with DOC; instead, DOC‐mediated reductions in resource availability seem to affect reproductive output by limiting postmaturation growth and ultimately maximum size. Further support for the idea of limited postmaturation growth in dark lakes comes from the fact that the relationship between initial growth and DOC is fairly weak (Fig. 1b); greater differences in size are only observed at later ages, after the fish have reached maturity (Fig. 1c). Larger female fish have been shown to be disproportionally valuable in fisheries due to their ability to produce greater numbers of eggs (Green, 2008), so the lack of large fish in high‐DOC environments may be contributing to a loss of reproductive output and potentially fishery productivity.

Resource limitation has been found to reduce maximum size and reproductive output in other systems and species. For example, Grether, Millie, Bryant, Reznick, and Mayea (2001) and Reznick, Butler, and Rodd (2001) found that guppies living in resource‐rich, low‐competition environments were able to grow faster, reach larger sizes, and allocate more resources to reproduction. Resource limitation has also resulted in smaller maximum sizes for other populations of bluegill (Aday, Philipp, & Wahl, 2006), as well as for yellow perch (Heath & Roff, 1996) and northern pike (Diana, 1987). As reproductive output is strongly related to body size in fishes (Roff, 1983), we could assume that these species were also limited in their reproductive potential. This evidence suggests that DOC‐mediated changes in resource availability and life history patterns resulting in reduced fecundity may occur in other fish species.

Low energy availability and/or high DOC levels have been shown to reduce initial growth in fish populations such as bluegill (Gerking, 1962), perch (Horppila et al., 2010), rainbow smelt (King, Shuter, & Zimmerman, 1999), and walleye and lake trout (Benoît, Beisner, & Solomon, 2016); however, we did not observe a strong relationship between DOC and initial growth in our study (Fig. 1b). We did, however, observe that fish in lakes at the darker end of the gradient grew more slowly at later ages, and this resulted in lower maximum sizes (Fig. 1a,c). Similarly, Rask and Tuunainen (1990) found that European perch and roach were smaller in length at a given age in darker Finnish lakes. This suggests that bluegill, and perhaps some other species, are not resource‐limited at small sizes across a DOC gradient but may become so in darker lakes as they get bigger and require more energy to maintain a larger body size as well as reproductive output, resulting in slower adult growth.

There was also a trend of later age at maturity in the darker lakes, suggesting that the DOC‐mediated resource limitation restricts the age at which females can produce eggs. This fits with established patterns as it has been shown that resource limitation can delay maturity as females must wait longer until they have amassed sufficient body mass and energy reserves to make reproduction viable (Drake et al., 1997; Roff, 1982; Stearns & Koella, 1986; Tyler & Dunn, 1976). Interestingly, despite bluegill in darker waters maturing later, maximum age and number of spawning years showed no trend with DOC, suggesting that even though fish mature later, it does not necessarily reduce the number of spawning years. This again reinforces the importance of maximum body size and limitation of postmaturation growth, as even though bluegill from high‐DOC lakes may live and/or spawn for equivalent periods to low‐DOC populations, they are still limited in their potential and realized reproductive output.

Our results suggest that life history parameters are more constrained at the higher end of the DOC gradient and more flexible in clearer waters. This is true for growth and for size and age at maturity, suggesting that fish in clear waters can take advantage of several different strategies such as growing fast, maturing early, and dying relatively young, or growing more slowly, maturing later and larger, and living longer. On the other hand, fish in darker waters can only grow relatively slowly and mature relatively late; they can live for a variable amount of time, but never reach the large sizes seen in the clearer lakes. Similar wedge‐shaped, or unimodal, patterns of biomass and production across DOC gradients have been observed in other studies of primary producers, primary consumers, and fishes (Craig et al., 2015; Karlsson et al., 2015; Kelly et al., 2014; Seekell et al., 2015a). Some of this variation may be explained through the dynamics of nutrient and light limitation, along with thermocline depth (Seekell, Lapierre, & Karlsson, 2015b; Solomon et al., 2015). In clear waters with deep thermoclines and light penetration, there may be high variability in nutrient concentrations which may stimulate or constrain growth and productivity with a minimal effect of light (Fig. 4a, Seekell et al., 2015b). However, darker lakes may reach a threshold of light limitation, and habitat availability for primary production (particularly for benthic processes), after which an increase in nutrients makes little difference to areal productivity (Craig et al., 2015; Godwin et al., 2014; Seekell et al., 2015b; Solomon et al., 2015). The surface layers of high‐DOC lakes may be highly productive (e.g., Fig. 4b), but the restricted volume of the epilimnion means that productivity is limited at the whole lake level. The potential for variability in clearer lakes explains why some of our results (e.g., initial growth and age at maturity) showed trends, but were not significant. Bluegill in clear lakes are subjected to a gradient of productivity levels which allow for variation in life history strategies, whereas in the darker lakes, fish growth and thus other life history characteristics are constrained by low levels of areal productivity. This may make it easier to predict how fish populations may respond to increasing DOC, but less so in situations where DOC levels are decreasing (e.g., Schindler, Curtis, Parker, & Stainton, 1996).

### 4.2 Potential confounding variables across lakes

While this study focuses on the effects of DOC on fish life histories, there may be other variables that can influence the productivity of fish across the DOC gradient. Density‐dependent competition can have a negative effect on fish growth and productivity (Rose, Cowan, Winemiller, Myers, & Hilborn, 2001; Ylikarjula, Heino, & Dieckmann, 1999); however, our results suggest that adult densities are similar across the DOC gradient and may actually be slightly lower in the darker lakes, which would relieve some of the competitive pressure for resources (Fig. 4c,d). High nutrient availability, and primary production, can also have positive effects on fish productivity (Downing & Plante, 1993). However, we found no difference in total phosphorous concentrations along the DOC gradient, and generally higher levels of epilimnetic chlorophyll *a* in the darker lakes where fish growth and reproductive output were lowest (Fig. 4a,b), suggesting that the high availability of phytoplankton in the photic layer did not compensate for the reduction in habitat and secondary resource availability in these lakes.

### 4.3 Implications for fish populations

Fishing pressure is another factor that can strongly influence fish life histories (Drake et al., 1997; Law, 2000), and it had a strong negative effect in the two most heavily fished lakes in this study. Allequash and Big Arbor Vitae were the only two lakes with high fishing pressure, and they stood out as having fast growth rates and higher mortality rates than the other lakes. In these lakes, fish had high potential to produce eggs, but were often harvested before they had a chance to do so; thus, their realized fecundity was much lower than their potential fecundity (Fig. 2d). These two populations had relatively high intrinsic rates of increase (*r *>* *1.48), which suggests that they are better able to withstand fishing pressure. However, the fish populations at the higher end of the DOC gradient tended to have lower *r* values, indicating that these populations may be less resilient to sustained fishing pressure. In addition, populations with the life history characteristics common to darker lakes (e.g., slower growth and later age at maturity) tend to be more susceptible to fishing pressure (Jennings, Reynolds, & Mills, 1998), increasing the likelihood that they may collapse, and may do so under lower levels of fishing pressure than in many clearer lakes. Modeling the effects of increased fishing pressure on the resilience of fish populations in darker lakes could be beneficial and allow the tailoring of fisheries management strategies both spatially and under future browning scenarios (Jennings et al., 1998).

## 5 CONCLUSIONS

Dissolved organic carbon has many complex physical and biological effects on lakes, and understanding how DOC concentrations impact fish ecology is crucial in order to predict how ecosystems work both spatially, and as lakes get browner. DOC‐mediated resource limitation appears to reduce postmaturation growth rates and thus size at age of bluegill in darker lakes, which in turn decreases lifetime fecundity. This could be a major factor in the reduction of fish productivity that has been observed with DOC in previous studies (Finstad et al., 2014; Karlsson et al., 2009) and provides new mechanisms to the emerging framework of how DOC affects aquatic ecosystem productivity.

## ACKNOWLEDGEMENTS

Funding was provided by the Natural Sciences and Engineering Research Council of Canada. The staff of the University of Notre Dame Environmental Research Center facilitated our field work there. Technical assistance was provided by Pierre‐Olivier Benoit, Ludovick Brown, Patrick Kelly, Jacob Lerner, Karling Roberts, Greg Sass, Jacob Ziegler, and Jacob Zwart. Members of the Wisconsin DNR, several anonymous reviewers, as well as Andrew Hendry and Melissa Lenker provided useful comments that improved the execution of this paper. Ethical approval was granted by the McGill University Animal Care Committee (2011‐5986), and collectors permits were granted by the Wisconsin, and Michigan DNR (SCP‐NOR‐341‐0513 & SCP‐NOR‐341‐0514). Any use of trade, firm, or product names is for descriptive purposes only and does not imply endorsement by the U.S. Government.

## AUTHOR CONTRIBUTIONS

All authors conceived of and designed the study. NC collected the data. NC and CS analyzed the data. NC wrote the manuscript, and all authors contributed to revisions.
