# Supplementary material for: Life history constraints explain negative relationship between fish productivity and dissolved organic carbon in lakes
Source: Ecol Evol. 2017 Jul 3;7(16):6201–9. doi: 10.1002/ece3.3108 (PMC5574801; doi:10.1002/ece3.3108)

**Appendix S1. Data on number of fish available for various analyses**

| lake | n bluegill measured (F & M) | n bluegill gonads examined (F) | n gonads for maturity analysis (F) | n gonads used for egg analysis (F) | n otoliths analysed (F/M) |
| --- | --- | --- | --- | --- | --- |
| Big Arbor Vitae | 368 | 104 | 32 | 24 | 67 (51/16) |
| Crampton | 311 | 92 | 40 | 27 | 53 (38/15) |
| Allequash | 222 | 53 | 53 | 14 | 54 (36/18) |
| Erickson | 160 | 72 | 43 | - | 29 (19/10) |
| Bay | 214 | 79 | 25 | 35 | 50 (32/17)* |
| Deadwood | 209 | 71 | 35 | 23 | 39 (30/9) |
| Tenderfoot | 222 | 67 | 27 | 24 | 47 (30/16)* |
| Birch | 151 | 55 | 40 | - | 36 (18/18) |
| McCullough | 58 | 24 | 11 | 7 | 27 (21/6) |
| Red Bass | 271 | 75 | 26 | 22 | 40 (31/9) |
| Hummingbird | 93 | 38 | 20 | 6 | 44 (27/17) |

F = female, M = male. *n bluegill measured* refers to total number of bluegill collected in fyke nets for which we obtained length-weight data, whether they were euthanised or released. *n bluegill gonads examined* refers to the total number of females for which we collected gonad weight from. *n gonads for maturity analysis* refer to the number of gonads used in the size at maturity analysis – i.e. from the date that gonad mass was at its peak in each lake. Asterisk (*) indicates that there were fish in this group for which sex could not be determined.

**Appendix S2: Supplementary figures of lake level fish data.**

Lake code key: BV – Big arbor Vitae, CR – Crampton, AQ – Allequash, ER – Erickson, BA – Bay, DW – Deadwood, BH – Birch, TF – Tenderfoot, MC – McCullough, RS – Red Bass, HB – Hummingbird.

**Figure 2a**. Von-Bertalanffy growth curves fit to female length at age data (black points and lines), as well as combined female and male data (black and grey points, grey lines) generated from otolith analysis of a subset of fish collected from each lake. Lakes are in order of increasing DOC.

**Figure 2b**. Female bluegill age determined by otolith analysis as a function of fish weight. Lakes are in order of increasing DOC.

**Figure 2c**. Gonad mass of pre-spawn females plotted against body length. Lakes are in order of increasing DOC. These data were used to calculate size at maturity, as the break point between the slope of the regression lines for immature and mature fish. The model did not fit well to the Tenderfoot Lake (TF) data, for which the estimated size at maturity may therefore be too low. There were no immature fish collected in Deadwood Lake (DW) so the immature gonad mass for this lake was estimated as an average of the other lakes.

**Figure 2d**. Female gonad mass as a function of fish mass. Lakes are in order of increasing DOC. This figure is for female bluegill collected just before spawning.

**Figure 2e**. Number of eggs per gonad as a function of gonad mass for female bluegill just before spawning. Lakes are in increasing order of DOC.

**Figure 2f**. Egg number per gonad as a function of fish length. Lakes are in order of increasing DOC. Egg number was significantly related to fish length, but not DOC (LME length slope: 0.73 ± 0.3, DOC slope: -0.01 ± 0.24).

**Figure 2g**. Egg width as a function of fish length. Lakes are in order of increasing DOC. Egg width was not significantly related to fish length or DOC for 8 out of 9 lakes examined (LME length slope: 0.3 ± 0.3, DOC slope: -0.16 ± 0.24, with the exception being Red Bass (RS) Lake.

**Figure 2h**. Lake specific bluegill length-frequency relationships. Note that McCullough Lake was sampled by angling, rather than fyke net. Lakes are in order of increasing DOC.


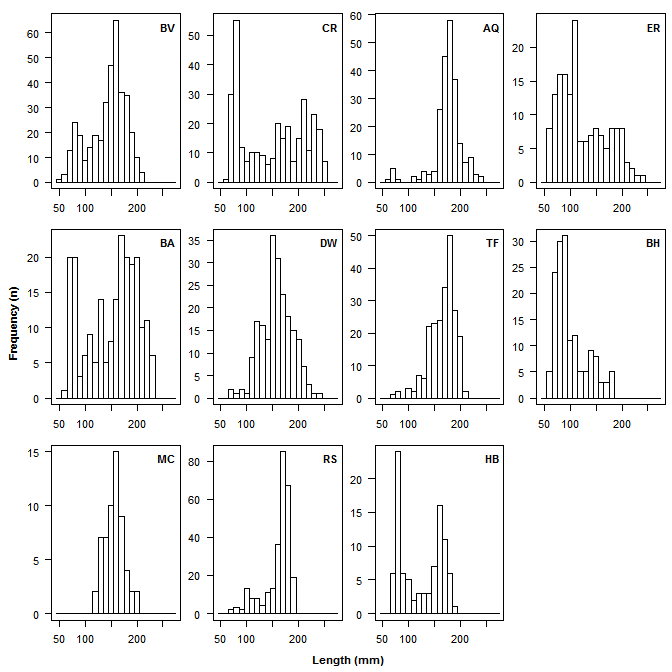

Supplement: Supplementary file 1 [file ECE3-7-6201-s001.docx]
